# Enterprise in American Hospital Management

**Published:** 1915-05-08

**Authors:** 


### ENTERPRISE IN AMERICAN HOSPITAL MANAGEMENT.

### The Hutchinson Museum and its Lessons.

It can hardly be without regret that many British practitioners will learn that the clinical museum established by the late Sir Jonathan Hutchinson is to be lost to this country. An arrangement has been made, we understand, for the transference of the museum to the Johns Hopkins Hospital; and as fate decrees its loss to London, we could not wish it a more worthy home than the great post-graduation school of the United States. It is not unwelcome to us to know that so characteristic a memorial of the work of a great British clinician should be established on the other side of the Atlantic and in association with a scheme having aims which the Hutchinson Museum from its very beginnings was intended to promote. Though we are keenly conscious of a sense of loss, and realise that things might have been otherwise, we cultivate no grudge against our American *confrères*. Rather we congratulate them on a decided addition to the value and interest of their medical and educational machinery, and we are sure they will honour the museum as it merits to be honoured and will use it for the best and most worthy ends.

### The Museum's Motive.

The motive of the museum, as many of our readers must know, was to illustrate by drawings, paintings, and photographs all clinical conditions capable of being thus represented. With sustained and unfailing industry, and with unwearied interest, Hutchinson, during a long course of years, collected material from the abundant stores of his hospital and personal practice, and he spared no expense in obtaining the artistic help which was necessary. His outlook and sympathies, as is well known, were of a most catholic order, and he made no pretence to exclude from his purview anything which would help to the spread of clinical truth and to a solution of the problems of disease. Further, he believed intensely in the educational value and purpose of museums. Hence his collection was not limited to one particular department of practice, nor was his object the display of mere curiosities and eccentricities. On the contrary, recognising that bedside teaching is necessarily conditioned by limited opportunities, he intended his museum to provide pictorial illustrations of cases allied to any one with which the clinical teacher may at the moment be concerned. Thus the lesson was to be set forth on broader lines than those of the individual patient, and this, not in mere words, but in pictures drawn from life. This was the practice he adopted in his own clinical demonstrations, and at least his audiences realised its value. Indeed, for anyone who has seen the method in operation, it is a matter for wonder that it is so little utilised. The clinical teacher is constantly hampered by the lack of sufficient cases of one and the same order, so that he has not the advantage of comparison and contrast. With a clinical museum or picture gallery at hand this deficiency is largely overcome, and teaching so illustrated is enormously increased in range and value. Thus the clinical museum, in addition to the opportunities which it offers for direct study, is a most effective auxiliary to bedside instruction, and though few may be able to use it with the genius of Hutchinson, no teacher could fail to find it both helpful and stimulating.

### Patients or Pictures?

If the principle above stated is sound, and if the Hutchinson collection was a highly effective expression of it, how does it come about that London, with its boasted clinical schools, has allowed the museum to escape across the seas? The first reason, we are afraid, is, that the true value of the museum as an educational instrument is but imperfectly appreciated. Patients, it is insisted, and not pictures, are the true texts of the clinical teacher. We quite agree that patients must occupy the first place, but this does not prove that pictures are useless. Our contention is that the purpose of the pictures is not to replace the patients, but to supplement them. No doubt they would be unnecessary if the hospital service were able at the desired moment to supply a number of cases of the same order but illustrating different features of the disease under discussion. But notoriously this is a rare and exceptional event. Can compensation for the defect be secured? There is only one way of securing it—namely, to have at hand a number of pictorial illustrations taken from actual cases, and acting, at least in some measure, as substitutes for these cases. This, in a word, is the adoption of Hutchinson's principle in the shape of the clinical museum.

### Medical and Clinical Pictures.

When value is not recognised, money is, of course, hard to obtain. Thus it came about that in its earlier years, not only the making of the collection, but its housing and exhibition were conducted at the personal cost of the founder. Afterwards some co-operation was secured, and a special building was erected for the museum, but no fund was established for its maintenance, and on Sir Jonathan's death it was left derelict. At last it has become associated with a well-established organisation, and there doubtless it will fulfil the purposes for which it was intended. The moral would seem to be that our medical schools ought to be led to realise that their machinery would be very greatly improved by the organisation of a clinical museum or picture gallery. Made by co operative effort continued through several generations, such an institution would be of enormous benefit to the student, as also to the practitioner returning for the revival and reinforcement of his knowledge. The pathological museum is regarded as part and parcel of the hospital equipment, and we have no desire to discourage it. But in a scheme of teaching the art of medicine pictures of clinical and diagnostic significance are surely not less important than collections of *post-mortem* specimens. Were these pictures arranged in connection with a medical school the expense entailed would necessarily be much less than in the case of a museum dependent mainly on individual effort. The teaching and example of Sir Jonathan Hutchinson are likely long to endure in medical history. To us his attitude towards museums was among his wisest influences. Possibly his countrymen may learn to appreciate this attitude now they know that its value is recognised elsewhere.

